# Predicting Chemo-Radiotherapy Sensitivity With Concordant Survival Benefit in Non-Small Cell Lung Cancer *via* Computed Tomography Derived Radiomic Features

**DOI:** 10.3389/fonc.2022.832343

**Published:** 2022-06-22

**Authors:** Yixin Liu, Haitao Qi, Chunni Wang, Jiaxing Deng, Yilong Tan, Lin Lin, Zhirou Cui, Jin Li, Lishuang Qi

**Affiliations:** ^1^ College of Intelligent Systems Science and Engineering, Harbin Engineering University, Harbin, China; ^2^ Basic Medicine College, Harbin Medical University, Harbin, China; ^3^ College of Bioinformatics Science and Technology, Harbin Medical University, Harbin, China

**Keywords:** computed tomography, non-small cell lung cancer, concurrent chemo-radiotherapy, radiomic signature, candidate therapeutic agents

## Abstract

**Background:**

To identify a computed tomography (CT) derived radiomic signature for the options of concurrent chemo-radiotherapy (CCR) in patients with non-small cell lung cancer (NSCLC).

**Methods:**

A total of 226 patients with NSCLC receiving CCR were enrolled from public dataset, and allocated to discovery and validation sets based on patient identification number. Using CT images of 153 patients in the discovery dataset, we pre-selected a list of radiomic features significantly associated with 5-year survival rate and adopted the least absolute shrinkage and selection operator regression to establish a predictive radiomic signature for CCR treatment. We performed transcriptomic analyzes of the signature, and evaluated its association with molecular lesions and immune landscapes in a dataset with matched CT images and transcriptome data. Furthermore, we identified CCR resistant genes positively correlated with resistant scores of radiomic signature and screened essential resistant genes for NSCLC using genome-scale CRIPSR data. Finally, we combined DrugBank and Genomics of Drug Sensitivity in Cancer databases to excavate candidate therapeutic agents for patients with CCR resistance, and validated them using the Connectivity Map dataset.

**Results:**

The radiomic signature consisting of nine features was established, and then validated in the dataset of 73 patients receiving CCR log-rank P = 0.0005, which could distinguish patients into resistance and sensitivity groups, respectively, with significantly different 5-year survival rate. Furthermore, the novel proposed radiomic nomogram significantly improved the predictive performance (concordance indexes) of clinicopathological factors. Transcriptomic analyzes linked our signature with important tumor biological processes (e.g. glycolysis/glucoseogenesis, ribosome). Then, we identified 36 essential resistant genes, and constructed a gene-agent network including 10 essential resistant genes and 35 candidate therapeutic agents, and excavated AT-7519 as the therapeutic agent for patients with CCR resistance. The therapeutic efficacy of AT-7519 was validated that significantly more resistant genes were down-regulated induced by AT-7519, and the degree gradually increased with the enhanced doses.

**Conclusions:**

This study illustrated that radiomic signature could non-invasively predict therapeutic efficacy of patients with NSCLC receiving CCR, and indicated that patients with CCR resistance might benefit from AT-7519 or CCR treatment combined with AT-7519.

## Introduction

Lung cancer was the first most commonly diagnosed cancer (1), with non-small cell lung cancer (NSCLC) accounting for 80% to 85% of cases (2). More than 30% of patients with NSCLC have locally advanced and unresectable disease, and the 5-year survival rate is less than 15% (3). International guidelines recommend concurrent chemo-radiotherapy (CCR) as a standard first-line treatment option for locally advanced stage NSCLC patients (4). However, the prognosis achieved with CCR remains unsatisfactory with the 5-year survival rate of less than 25% (5, 6). Therefore, it is imperative to develop a clinically feasible signature to stratify patients who might benefit from CCR treatment, avoiding these side effects of unnecessary treatment.

Currently, most predictive signatures for CCR therapeutic efficacy were constructed based on molecular characterization using genomic and proteomic technologies (7–9). However, these techniques are limited due to tumors are spatially and temporally heterogeneous, which could not provide a complete characterization of the tumor (10). In contrast, medical imaging can be used to non-invasively and cost-effectively visualize the characteristics of entire tumor, providing dynamic information that can be used to monitor the occurrence and development of tumors (11, 12). Currently, computed tomography (CT), which is the most commonly used imaging modality in oncology, especially lung cancer, allows non-invasive detection of tissue density and describes tumor spatial heterogeneity (12).

Radiomics converts medical images into high-throughput quantitative features; this is a new field that could be the vanguard of precision medicine (10, 13), which offers the possibility to minimize adverse effects and optimize the efficacy of treatments (14). Current, most researchers firstly developed prognostic signatures (15–17) for patients not receiving CCR and then demonstrated that only the high-risk patients predicted by the signatures showed significantly survival benefit after CCR treatment. Obviously, such prognostic signatures were just able to identify patients with poor prognosis who need CCR treatment, but unable to identify patients who might be sensitive to treatment. In order to provide support in patient management and achieve maximum clinical benefit, the development of CT derived radiomic signature for predicting the patients sensitive to CCR need to be assessed to predict the therapeutic efficacy of CCR treatment.

In this study, using CT images of patients, we develop a non-invasive radiomic signature for patients with locally advanced stage NSCLC receiving CCR, which might help to accurately predict therapeutic efficacy for CCR treatment with improved 5-year survival rate. Subsequently, based on the dataset with matched CT images and gene expression profiles, we characterized the underlying functional pathways reflected by the radiomic features in the signature and tentatively captured the potentially beneficial agents required for the treatment of patients with CCR resistance based on cancer cell lines dataset.

## Materials and Methods

### Data Sources

In this study, the NSCLC-Radiomics (NR) dataset (10) with DICOM CT scans was downloaded from The Cancer Imaging Archive (TCIA, https://www.cancerimagingarchive.net/, 2020), including 422 patients previously treated with CCR or radiotherapy. The inclusion criteria of the samples for CCR treatment planning were as follows: 1) available treatment-naive CT scans; 2) confirmed NSCLC; 3) patients treated with CCR; 4) available survival information. Finally, 226 patients with locally advanced stage NSCLC receiving CCR were preselected and divided into discovery (n = 153) and validation (n = 73) datasets based on patient identification number (pid), that is, the 153 patients whose pid wasn’t divisible by 3 were used as a discovery dataset to develop a radiomic signature for CCR treatment, and the remaining 73 patients were assigned to the validation dataset. These details and applications of the analyzed datasets are displayed in **Table 1** and **Supplementary Figure S1**.

**Table 1:** Baseline clinical characteristics of patients in the analyzed datasets.

	Discovery dataset (*n* = 153)	Validation dataset (*n* = 73)
**Age (years)**		
≤ 65	78 (51.0%)	35 (47.9%)
> 65	70 (45.8%)	34 (46.6%)
**Gender**		
Female	54 (35.3%)	28 (38.4%)
Male	99 (64.7%)	45 (61.6%)
**TNM stage**		
I	–	–
II	–	–
III	153 (100%)	73 (100%)
**T stage**		
T1	20 (13.1%)	20 (27.4%)
T2	68 (44.4%)	24 (32.9%)
T3	21 (13.7%)	10 (13.7%)
T4	41 (26.8%)	19 (26.0%)
**N stage**		
N0	–	–
N1	–	–
N2	97 (63.4%)	44 (60.3%)
N3	56 (36.6%)	29 (39.7%)
**Histologic subtype**		
ADC	18 (11.8%)	11 (15.1%)
SCC	53 (34.6%)	22 (30.1%)
LCC	53 (34.6%)	23 (31.5%)
NOS	22 (14.4%)	13 (17.8%)
**Average survival (Month)**	28.65	34.78

ADC, Adenocarcinoma; SCC, Squamous cell carcinoma; LCC, Large-cell lung carcinoma; NOS, Not otherwise specified subtype.

NSCLC-Radiogenomics (NRG) dataset (18) with DICOM CT scans and matched gene expression profiles were downloaded from TCIA, including 67 NSCLC patients treated with different therapeutic strategies, which was used to understand the biological processes linked to the radiomic signature.

Genome-wide CRISPR screening of NSCLC cells (n = 87) was downloaded from the DepMap portal (https://depmap.org/portal/download/; 2019). Dependency scores for around 17,000 candidate genes were calculated using the CERES algorithm (19). Essential genes for NSCLC were defined as the genes with a CERES score of <−1 across 75% of NSCLC cell lines. The DrugBank database (https://go.drugbank.com/) was used to identify therapeutic agents targeting essential genes.

Genomics of Drug Sensitivity in Cancer dataset (20) (GDSC, https://www.cancerrxgene.org, release-8.2), which contains responses to 345 anticancer agents across 917 cancer cell lines, with gene expression profiles and half-maximal inhibitory concentrations (IC_50_) values of cell lines were used to identify potential therapeutic agents.

Gene expression profiles of 27 samples treated with different concentrations (0.01 - 10 µM) of AT-7519 for 24 hours and the corresponding 124 untreated control samples were downloaded from Connectivity Map dataset (21) (CMap, https://clue.io/data/CMap2020#LINCS2020), including 12,328 genes. These samples were derived from A549 and HCC515 NSCLC cell lines. The samples treated with AT-7519 were divided into three dose groups: Low (dose < 1 µM), Middle (1 < dose < 10 µM) and High (dose = 10 µM), which was used to validate therapeutic efficacy of AT-7519.

### Image Segmentation and Radiomic Feature Extraction

The regions of interest (ROI) of CT scans in the NR and NRG datasets were publicly available. In general, the three-dimensional radiomic features that enabled quantification of the tumor characteristics were divided into ten groups according to the following: I) Tumor intensity, II) Shape, III) Texture, IV) wavelet filters, V) Laplacian of Gaussian filters, VI) Logarithm filters, VII) Square filters, VIII) Exponential filters, IX) Gradient filters and X) Squareroot filters features. For the NR and NRG datasets, radiomic feature extraction was performed for each CT scan with ROIs using free and open-source PyRadiomics (v2.2.0) libraries. An extraction intensity bin width was set at 25 HU and the slice thicknesses of all scans were interpolated to a voxel size of 1×1×1 mm^3^. The quantitative values of 1781 radiomic features (**Supplementary Table S1**) were calculated according to feature definitions in the PyRadiomics documentation (https://pyradiomics.readthedocs.io/en/latest/index.html) by the Imaging Biomarker Standardization Initiative (22). 

### Construction of the Radiomic Signature for CCR Treatment

In the discovery dataset, radiomic features whose quantitative values were significantly associated with the 5-year survival rate were identified as CCR-associated features. Based on the CCR-associated features, we adopted the least absolute shrinkage and selection operator (LASSO) regression (23) using “glmnet” R package to establish an optimal predictive model, and defined it as a predictive radiomic signature for CCR treatment. “Cox” was set as the family in the model. Ten-fold cross-validation was performed using cv.glmnet function to select lambda minimum to give the minimum cross-validated error. The resistant score of the signature for each patient was calculated *via* a linear combination of features in the signature that were weighted by their respective coefficients as follows:


$$\text{Risk score}=\sum_{i=1}^{n}w_{i}FeatureValue_{i}\;i\in \;n$$

where *i* represents the *i-th* feature in the signature; *w_i_* represents the weight of the *i-th* feature derived from LASSO model; *FeatureValue_i_* represents the quantitative value of the *i-th* feature; and *n* represents the number of features contained in the signature.

The median value of resistant scores of the radiomic signature in the discovery dataset was used as the cut-off value for dividing patients into the resistance (≧ Median) and sensitivity (< Median) groups.

### Statistics Analyzes

The 5-year survival rate of the patients was used as the end point of interest. Patients with more than 5 years follow-up were censored at 5 years because deaths occurring past five years were not likely to be related to CCR treatment. Survival curves were estimated using the Kaplan-Meier method and statistically compared using the log-rank test (24). To analyze the associations between the different influencing factors and the 5-year survival rate of the patients, a univariate Cox regression model was used, and to test the independent association of the radiomic signature with the 5-year survival rate after adjusting for the clinical parameters recorded in the data, a multivariate Cox regression model was used. Hazard ratio (HR) and 95% confidence interval (CI) were generated using the Cox proportional hazards models, and the concordance index (C-index) (25) was also used to estimate the predictive performance of clinical factors. Time-dependent receiver operating characteristic curve (ROC) analysis (26) and the area under the curve (AUC) were performed to evaluate the radiomic signature’s performance in predicting the 1-, 3- and 5-year survival rates.

To assess the complementary effect of the radiomic signature on the clinical model in predicting the therapeutic efficacy in patients with NSCLC receiving CCR treatment, a radiomic nomogram was constructed using multivariate linear regression analysis (“rms” R package). Additionally, the predictive performance of the radiomic nomogram was evaluated based on C-index, calibration curve, and the decision curve analysis. The net reclassification improvement (NRI) (27) index was determined to quantify the radiomic signature’s incremental improvement using the “nricens” R package.

Spearman’s rank correlation was applied to investigate the association between the radiomic signature and clinical parameters. The “clusterProfiler” R package was used to conduct the functional enrichment analysis of the genes that were correlated with the radiomic features based on the current Kyoto Encyclopaedia of Genes and Genomes (KEGG) database, wherein a hypergeometric test was employed.

ESTIMATE (28) was introduced to estimate the immune score for a given sample by performing ssGSEA (29), based on its mRNA expression profiles using an “estimate” R package. The ssGSEA was also utilized to quantify the relative infiltration levels of 28 immune cell types in the tumor microenvironment by using a “GSVA” R package. The relative infiltration levels of each immune cell type were represented by an enrichment score in the ssGSEA analysis.

Student’s *t*-test was used to examine the intergroup difference by comparing samples treated with potential therapeutic agents and the corresponding untreated control samples. Binomial distribution was used to examine the difference in the distribution of the down-regulated and up-regulated resistance genes induced by the potential therapeutic agents.

Statistical analyzes were performed using R, version 3.5.3; *P* values were adjusted using the Benjamini-Hochberg procedure for multiple tests to control the false discovery rate (FDR). Statistical significance was defined as two-sided *P*<0.05 or FDR<0.05 for multiple tests.

## Result

### Construction and Validation of a Radiomic Signature for CCR Treatment

**Figure 1:**
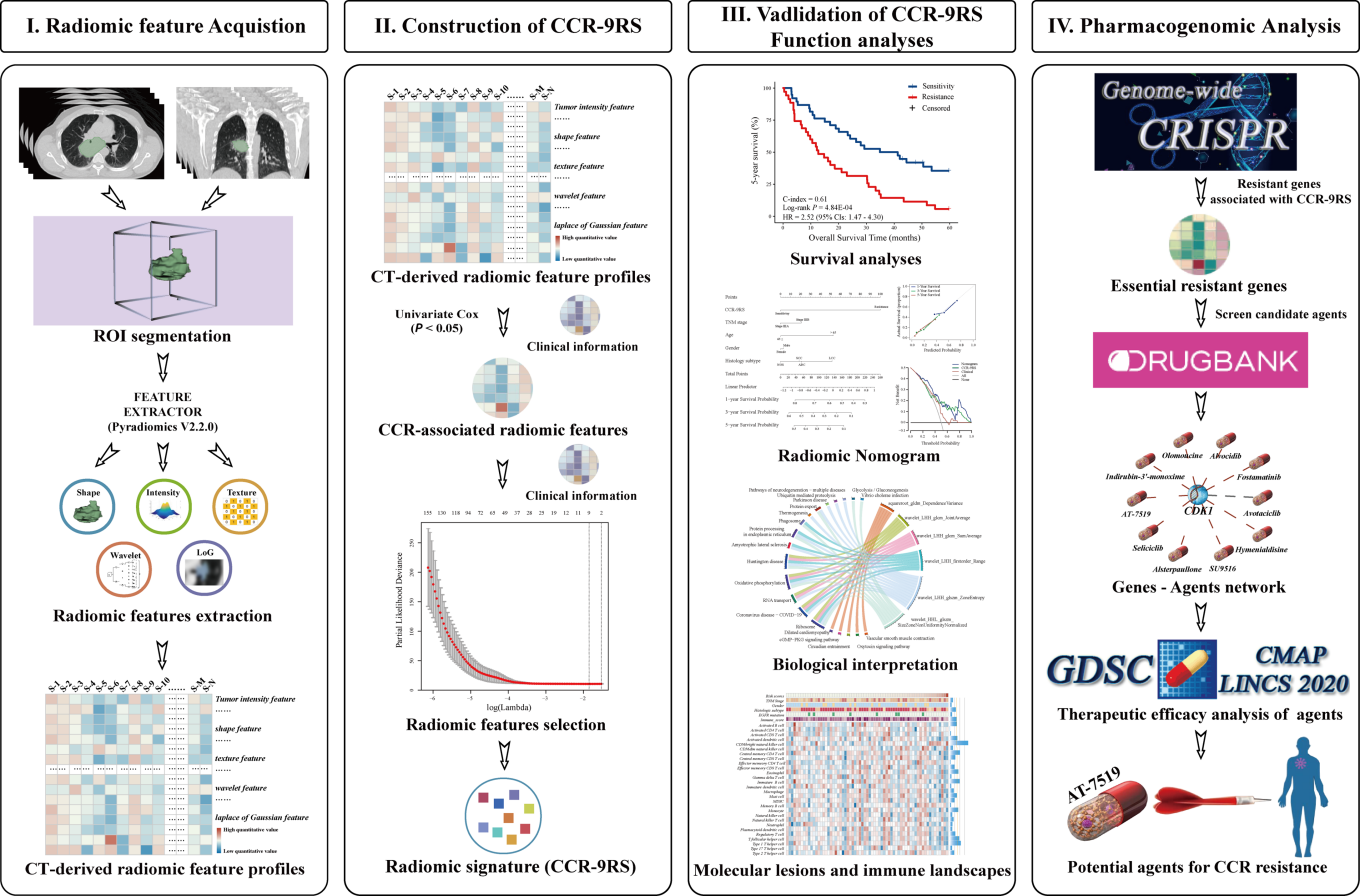
Flowchart of developing and validating of a radiomic signature derived from computer tomography (CT) for the patients with NSCLC receiving CCR treatment.

**Figure 2:**
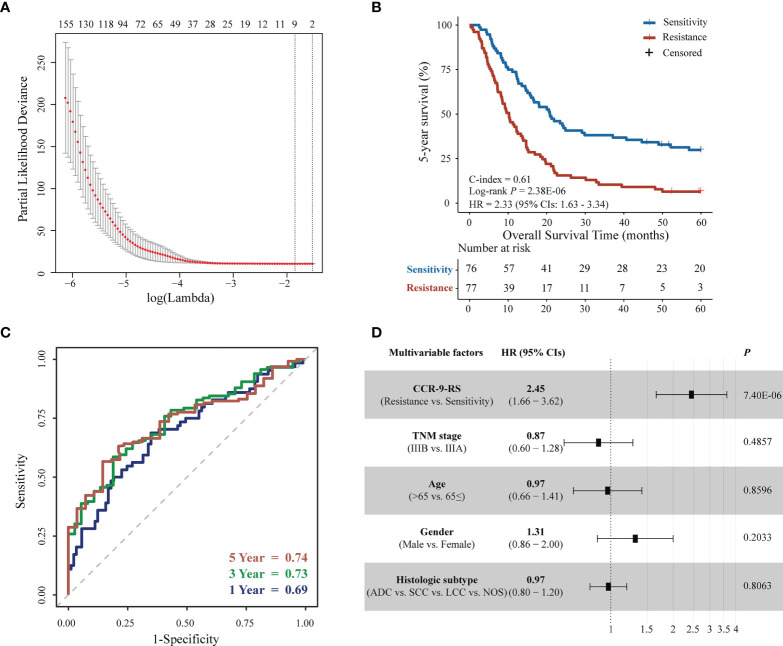
Feature selection and survival analyzes for patients with NSCLC receiving CCR in the discovery dataset. **(A)** Tuning parameter (λ) selection in the least absolute shrinkage and selection operator (LASSO) Cox model used a 10-fold cross-validation *via* minimum criteria. The area under the receiver operating characteristic (AUC) curve was plotted versus log(λ). **(B)** Kaplan–Meier curves of the 5-year survival rate for 153 patients. **(C)** Time-dependent receiver operating characteristic curve (ROC) of CCR-9RS in predicting the 1-, 3- and 5-year survival rates. **(D)** Multivariate Cox analyzes of CCR-9RS after adjusting for clinical factors.

**Figure 1** describes the flowchart of this study. In the discovery dataset, comprising 153 patients with NSCLC receiving CCR, we extracted 398 CCR-associated radiomic features which were potentially significantly associated with 5-year survival rate (Univariate Cox regression, *P* < 0.05). The CCR-associated features were selected as inputs for LASSO regression to generate a radiomic signature consisting of nine weighted features (denoted as CCR-9RS, **Figure 2A** and **Table 2**). The weighted sum of these nine radiomic features gave a resistant score for each sample (**Supplementary Table S2**). Using the median value (0.6241) of the 153 samples as the cut-off value, the patients were divided into resistance and sensitivity groups, respectively, with significantly 5-year survival rate differences (resistance vs. sensitivity = 77: 76, log-rank *P* = 2.38E-06, HR = 2.33, 95% CI: 1.63-3.34, C-index = 0.61, **Figure 2B**) in the discovery dataset. The time-dependent ROC curve of CCR-9RS in predicting the 1-, 3-, and 5-year survival rates were shown in **Figure 2C**, and the area under the curve (AUC) was 0.69, 0.73, and 0.74, respectively. In the multivariate Cox regression model, CCR-9RS remained significantly associated with the 5-year survival rate (P = 7.40E-06, HR = 2.45, 95% CI: 1.66-3.62, **Figure 2D**) after adjusting for TNM stage, age, gender and histologic subtype.

**Table 2:** Composition of CCR-9RS.

Radiomic feature name	HR	*P-*value	C-index
squareroot_gldm_DependenceVariance	1.06	0.0011	0.58
wavelet_LHH_glcm_JointAverage	1.10	0.0067	0.55
wavelet_LHH_glcm_SumAverage	1.05	0.0067	0.55
wavelet_LHH_firstorder_Range	1.01	0.0053	0.55
wavelet_LHH_glszm_ZoneEntropy	1.67	0.0051	0.56
wavelet_LLH_glrlm_LongRunHighGrayLevelEmphasis	1.01	0.0005	0.57
wavelet_LLH_glszm_SizeZoneNonUniformity	1.01	0.0002	0.58
wavelet_HHH_glszm_SizeZoneNonUniformity	1.02	2.91E-05	0.56
wavelet_HHL_glszm_SizeZoneNonUniformityNormalized	5370.36	0.0042	0.57

HR and P-value are the statistics calculated using a univariate Cox regression model. HR represents the risk coefficient of the quantitative values for the feature; P-value represents the significance of the quantitative values for radiomic feature.

**Figure 3:**
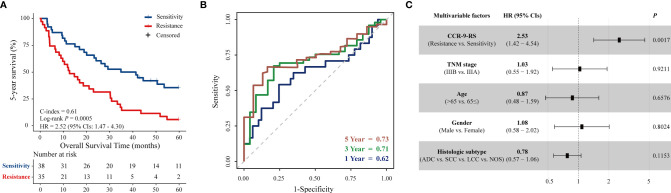
Validation of CCR-9RS. **(A)** Kaplan–Meier curves of 5-year survival rate for patients in the validation dataset (*n* = 73). **(B)** Time-dependent receiver operating characteristic curve (ROC) of CCR-9RS in predicting 1-, 3- and 5-year survival rates in the validation dataset. **(C)** Multivariate Cox analyzes of CCR-9RS after adjusting for clinical factors in the validation dataset.

The predictive performance of CCR-9RS was validated in the validation dataset, consisting of 73 patients with NSCLC receiving CCR. According to the trained cut-off (0.6241) of CCR-9RS, the 35 patients were classified into the resistance group, and exhibited significantly shorter 5-year survival rate than the 38 patients classified into the sensitivity group (log-rank P = 0.0005, HR = 2.52, 95% CIs: 1.47-4.30, C-index = 0.61, **Figure 3A**). The time-dependent ROC curve confirmed that CCR-9RS had a good performance for predicting 1-, 3- and 5-year survival rates in the validation dataset (**Figure 3B**). Multivariate Cox analysis revealed that 5-year survival rate was independently predicted by CCR-9RS after adjusting for the clinical factors in validation dataset (**Figure 3C**). Additionally, in order to exclude the influence of the not otherwise specified (NOS) subtype of NSCLC, CCR-9RS was also validated in the patients with clarifying histologic subtypes (Adenocarcinoma, Squamous cell carcinoma and Large-cell lung carcinoma) in the discovery dataset (*n* = 131, log-rank *P* = 4.81E-05, HR = 2.19, 95% CI: 1.49-3.22, C-index = 0.60, **Supplementary Figure S2A**) and validation dataset (*n* = 60, log-rank *P* = 0.0013, HR = 2.55, 95% CI: 1.42-4.61, C-index = 0.62, **Supplementary Figure S2B**), respectively.

### Incremental Value of CCR-9RS

**Figure 4:**
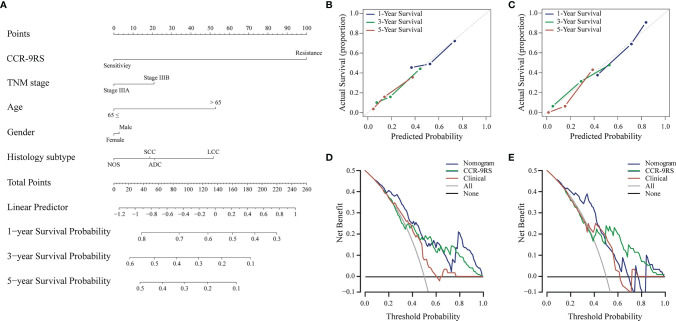
Radiomic nomogram and its performance for patients with NSCLC receiving CCR treatment. **(A)** Survival radiomic nomogram that incorporated with CCR-9RS and the clinical factors trained in the discovery cohort (*n*=153). The points of CCR-9RS and the clinical factors were obtained based on the top ‘points’ bar (scale: 0–100). The total point was calculated by summing the two points, and a line was drawn downward to the survival axes to determine the likelihood of 1-, 3-, or 5-year survival rate. **(B, C)** Calibration curves for the radiomic nomogram in the discovery and validation datasets; the diagonal gray line represents an ideal evaluation. **(D, E)** Decision curves for the radiomic nomogram in the discovery and validation datasets.

To further investigate whether CCR-9RS could provide incremental value for therapeutic evaluation of patients with NSCLC receiving CCR, we generated a radiomic nomogram (**Figure 4A**) that incorporated clinical factors (TNM stage, age, gender and histologic subtype) and CCR-9RS. The radiomic nomogram showed a significantly higher C-index relative to that of the clinical nomogram (**Supplementary Figure S3A**) and CCR-9RS alone based on the NRI index (*P* < 0.05, **Supplementary Figures S3B, C**) in the discovery dataset (C-index = 0.65, **Table 3**) and validation dataset (C-index = 0.66, **Table 3**). The calibration curves corresponding to the radiomic nomogram at 1-, 3-, and 5-year survival rates 7 showed good agreement between the estimations and the clinical outcomes in the discovery (**Figure 4B**) and validation datasets (**Figure 4C**). Furthermore, the decision curve analysis showed that the radiomic nomogram exhibited superior performance compared with the clinical nomogram across the majority of the range of reasonable threshold probabilities in the discovery (**Figure 4D**) and validation datasets (**Figure 4E**).

**Table 3:** Performances of different models.

	C-index (95% CIs)	
	Discovery dataset	Validation dataset
**Radomic nomogram**	0.65 (0.60 - 0.71)	0.66 (0.59 - 0.74)
**CCR-9RS**	0.61 (0.57 - 0.65)	0.61 (0.55 - 0.68)
**Clinical nomogram**	0.57 (0.51 - 0.63)	0.58 (0.50 - 0.66)

### Biological Function of CCR-9RS

**Figure 5:**
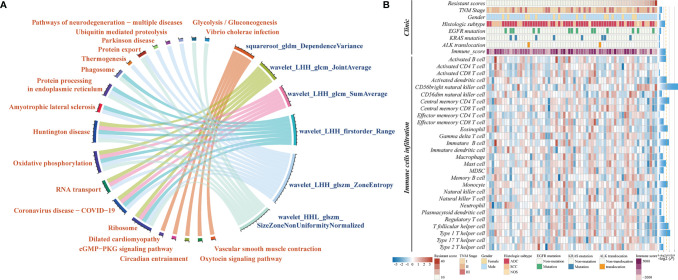
Molecular characteristics associated with CCR-9RS in NSCLC. **(A)** Gene-enrichment analysis of correlated genes with 6 radiomic features in CCR-9RS based on the Kyoto Encyclopedia of Genes and Genomes (KEGG) database in the NRG (NSCLC-Radiogenomics) dataset. **(B)** Molecular lesions and immune landscapes along with the resistant scores calculated by CCR-9RS. The correlation was estimated by Spearman rank correlation. The histogram on the right represents the significantly correlation with the resistant scores of CCR-9RS; the orange-dotted line represents *P* = 0.05.

The biological basis of CCR-9RS was evaluated in the independent NRG dataset (n = 67) with matched CT images and gene expression profiles. Using Spearman’s rank correlation analysis, we identified the significantly correlated genes of each feature in CCR-9RS (*P* < 0.05) and performed functional enrichment analysis for these correlated genes. It was observed that 6 of the 9 features were significantly enriched in 20 functional pathways (hypergeometric test, FDR < 0.05; **Figure 5A** and **Supplementary Table S3**), including “glycolysis/glucoseogenesis” (30), “ribosome” (31) and other functional pathways related to CCR treatment resistance. For example, we observed that “wavelet_HHL_glszm_SizeZoneNonUniformityNormalized” showed a strong positive correlation with genes enriched in “ribosome”, “glycolysis/glucoseogenesis” (**Supplementary Table S3**). The feature measures the variability of size zone volumes throughout the image, and a higher value of this feature represents a higher level of tumor heterogeneity, which might reflect the high glycolysis ability of a tumor with high CCR resistant capability (30, 32).

We also investigated the association of CCR-9RS with the molecular lesions (EGFR mutation, KRAS mutation and ALK translocation) and immune landscapes based on Spearman’s rank correlation analysis (**Figure 5B**). The resistant scores of CCR-9RS were not observed to be significantly associated with EGFR mutation (*P =* 0.3685), KRAS mutation (*P =* 0.8272) and ALK translocation (*P =* 0.6256). The result indicated that patients with CCR resistance might not benefit from the currently molecular-targeted therapies. Furthermore, we observed that patients with CCR resistance exhibited marginally significantly negatively correlated with immune scores (29) (*Rho* = -0.2222, *P =* 0.0707), and significantly negatively correlated with some immune cells (28), such as Activated dendritic cell (*Rho* = -0.2488, *P* = 0.0423), Activated B cells (*Rho* = -0.2387, P = 0.0517) and Central memory CD4 T cell (*Rho* = -0.2968, *P* = 0.0147). The result suggested that patients with CCR resistance had lower infiltration levels predicted by CCR-9RS, who might also not benefit from immunotherapy.

### Identification of Potential Therapeutic Agents for Patients Resistant to CCR Treatment

**Figure 6:**
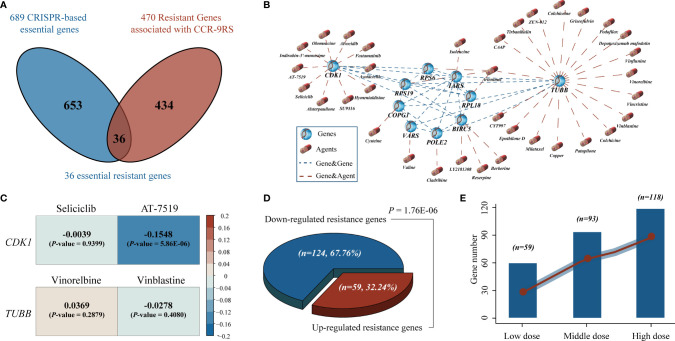
Identification of potential therapeutic agents for the patients resistant to CCR treatment. **(A)** Venn diagram of the resistant genes identified in tumor tissues (NSCLC-Radiogenomics dataset) and essential genes identified by CRISPR dataset. The blue circle represents the essential genes screened by CRISPR dataset and the red circle represents the resistant genes significantly positively associated with the resistant scores of the CCR-9RS in the NSCLC-Radiogenomics dataset. **(B)** A gene-agent network of essential resistant genes and candidate therapeutic agents using DrugBank database. The blue dotted line represents the significantly correlated essential resistant genes (Pearson correlation, FDR *<* 0.05, **Figure S4**) and the red dotted line represents the candidate therapeutic agents targeting essential resistant genes in DrugBank database. **(C)** The correlation analysis of four overlapped therapeutic agents corresponding to two essential resistant genes using GDSC cancer cell line dataset. **(D)** Binomial distribution for the down-regulated and up-regulated resistance genes induced by AT-7519. **(E)** The number of down-regulated resistance genes induced by AT-7519 in three dose groups (left to right: Low, Middle and High).

To further screen candidate therapeutic agents for patients resistant to CCR treatment, we first identified 470 resistant genes responsible for their resistance, whose expression values were significantly positively associated with the resistant scores of CCR-9RS in the NRG dataset (Spearman’s rank correlation, *Rho >* 0 and *P <* 0.05, **Figure 6A**). Second, we investigated genome-wide CRISPR-based loss-of-function screens derived from DepMap to pinpoint 689 essential genes for maintaining survival in 87 NSCLC cell lines and found 36 resistant genes to be essential genes for NSCLC. The correlations among ;the 36 resistant genes is displayed in **Supplementary Figure S4**. Therefore, the 36 essential resistant genes could be the potential targets of patients with CCR resistance. Third, taking advantage of the DrugBank database, we extracted 35 candidate therapeutic agents targeting 10 essential resistant genes and constructed a gene-agent network (**Figure 6B**). Finally, we input the 35 candidate therapeutic agents of gene-agent network into the GDSC cancer cell line dataset, and searched for 4 overlapped therapeutic agents (Seliciclib, AT-7519, Vinorelbine and Vinblastine) with completely IC_50_ values corresponding to two essential resistant genes (*CDK1* and *TUBB*). Using Spearman’s rank correlation analysis, we found that only the IC_50_ value of AT-7519 therapeutic agent was significantly negatively associated with the mRNA expression of the target gene (*CDK1*) in the GDSC dataset (*Rho* = -0.1548, *P* = 5.86E-06, **Figure 6C**). Therefore, AT-7519 was selected as a candidate therapeutic agent for the patients resistant to CCR treatment.

Finally, using the CMap dataset, we collected 27 samples treated with AT-7519 and the corresponding 124 untreated control samples to tentatively validate the therapeutic efficacy of AT-7519 for samples with CCR resistance. The detailed information of cell line samples treated with AT-7519 have been described in the **Supplementary Table S4**. Among the 341 resistant genes measured in the CMap dataset, we found that 183 resistant genes were significantly differently expressed between the AT-7519-treated and control groups (Student’s *t*-test, FDR < 0.05). Herein, 124 of the 183 resistance genes were significantly down-regulated induced by AT-7519, including the targeted *CDK1* gene of AT-7519 (Student’s *t*-test, *P* = 0.0046, **Supplementary Figure S5**), and showed a significant difference in the resistance genes distribution of the down-regulated induced (67.76%) and up-regulated induced (32.24%) by binomial distribution (*P* = 1.76E-06, **Figure 6D**). Furthermore, we divided the samples treated with AT-7519 into three dose groups: Low (*n* = 15), Middle (*n* = 8) and High (*n* = 4), and found that significantly more resistant genes (Low, 59; Middle, 93; High, 118, **Figure 6E**) were down-regulated induced by AT-7519, and the degree was gradually increased with the enhanced doses.

## Discussion

Radiomics is an emerging technique that converts traditional medical images into high-dimensional features, and has been widely applied in early diagnosis, prognosis and therapeutic efficacy evaluation, guiding clinicians develop individualized treatment plans for patients. In this study, we established a CT derived radiomic signature (CCR-9RS), which is the predictor of the therapeutic efficacy in patients with locally advanced stage NSCLC receiving CCR treatment. The radiomic signature successfully stratified NSCLC patients into the resistance and sensitivity groups with significantly different 5-year survival rate when they receiving CCR treatment. The combination of clinical factors with the radiomic signature in a radiomic nomogram could significantly improve the predictive performance of the clinical evaluation system in the discovery and validation datasets. These results indicated that CCR-9RS could provide additional predictive information for patients within the same clinical factors, and it will be worthwhile to develop this signature as a non-invasive predictive tool for clinical application.

Additionally, we tentatively estimated the predictive performance of CCR-9RS in early stage (stage I-II) NSCLC patients receiving radiotherapy, which is a guideline-recommended treatment for some early stage patients, based on the hypothesis that patients who were resistant to CCR should be resistant to both chemotherapy and radiotherapy. Here, we collected 93 stage I and 40 stage II NSCLC patients receiving radiotherapy in the NR dataset, and found that the resistance patients predicted by CCR-9RS also had significantly shorter 5-year survival rate than the predicted sensitivity patients (resistance vs. sensitivity = 55 vs. 78, log-rank *P* = 0.0138, HR = 1.61, 95% CI: 1.10-2.35, C-index = 0.57, **Supplementary Figure S6A**) when they receiving radiotherapy only. The time-dependent ROC curve confirmed the good performance of CCR-9RS in predicting 1-, 3- and 5-year survival rates of patients receiving radiotherapy (**Supplementary Figure S6B**). Multivariate Cox analysis revealed that 5-year survival rate was independently predicted by CCR-9RS after adjusting for the clinical factors in the radiotherapy dataset (**Supplementary Figure S6C**). This result indicated that CCR-9RS might also predict the efficacy of radiotherapy for early stage NSCLC patients, which needs further validation.

The underlying biological progression of the radiomic signature for CCR treatment is favorable for clinical application. Therefore, we first revealed that several known cancer-related functional processes, including “glycolysis/glucoseogenesis”, “ribosome” and other functional pathways related to CCR resistance, might be reflected by radiomic features in CCR-9RS. Next, we found that patients with CCR sensitivity were characterized by a higher immune score and levels of some immune cell infiltration (such as Activated dendritic cell and Activated B cells), providing evidence that patients sensitive to CCR treatment with higher infiltration levels might benefit from CCR treatment in the molecular mechanism. In contrast, patients with CCR resistance might not benefit from targeted therapy or immunotherapy, which requires further analysis of the benefit from other therapeutic agents.

In order to further screen potential therapeutic agents for the patients with CCR resistance, we first identified resistant genes significantly positively associated with the resistant scores of CCR-9RS in the tumor tissues (NRG dataset). Thereafter, *via* the leveraging the genome-scale CRIPSR data, we pre-selected a set of essential resistant genes, which could be the potential targets of the patients with CCR resistance. Then, we extracted candidate therapeutic agents targeting essential resistant genes and constructed a gene-agent network using the DrugBank database. Finally, we identified AT-7519 as a therapeutic agent for samples with CCR resistance in GDSC dataset. Furthermore, we tentatively validated the therapeutic efficacy of AT-7519 for samples with CCR resistance using the CMap dataset that significantly more resistant genes, positively correlated with the resistant scores of CCR-9RS, were down-regulated induced by AT-7519, and the degree was gradually increased with the enhanced doses of AT-7519. The results suggest that the patients with CCR resistance might benefit from AT-7519 or CCR treatment combined with AT-7519. We additionally explored the underlying correlation of AT-7519 and immunetherapy, and found that AT-7519 induced significant increases in the expression levels of 17 immune inhibitor/checkpoint genes (33) in CMap dataset (Student’s t-test, *P* < 0.05, **Supplementary Figure S7**), such as *CTLA4* (P = 0.0002), *PDCD1* (P = 0.0298) and *IDO1* (*P* = 6.61E-06). The correlation between AT-7519 and immunotherapy has not been mentioned yet, which merits further exploration. AT-7519 is an ATP competitive CDK inhibitor with effective anti-proliferative activity and has been undertaken or are undergoing the phase I and II clinical trials in a variety of solid tumors, including colorectal cancer (34), cervical cancer (35) and ovarian cancer (36). Therefore, AT-7519 would be a practical therapeutic agent for NSCLC patients with CCR resistance, because the conventional drug in new use can avoid the time-consuming and expensive procedure of new drug development (37).

This study still had some limitations. First, as a single-center retrospective study, the predictive estimation of CCR-9RS still need to be further validated in multicenter clinical trial studies. Second, our study indicated that the NSCLC patients with CCR resistance might benefit from AT-7519 or CCR treatment combined with AT-7519, which should be further validated in the phase of clinical development for cancer treatment.

In conclusion, the radiomic signature developed in this study could be applied to identify patients with NSCLC, who might benefit from CCR treatment prior to treatment, thus allowing clinicians to monitor the progress of patients. Furthermore, AT-7519 was captured as a potentially therapeutic agent for NSCLC patients with CCR resistance, which is worth exploring in future studies.

## Data Availability Statement

The datasets for this study can be found in the NSCLC-Radiomics and NSCLC-Radiogenomics datasets (TCIA, https://www.cancerimagingarchive.net/, 2020), the Genome-wide CRISPR (https://depmap.org/portal/download/; 2019), the GDSC dataset (https://www.cancerrxgene.org, release-8.2) and the CMap dataset (https://clue.io/data/CMap2020#LINCS2020).

## Ethics Statement

All data in this study source from public data sets. The patients/participants provided their written informed consent to participate in this study.

## Author Contributions

Experiment conceiving, LQ and JL. Manuscript writing, LQ and YL. Results interpretation, JL, LQ, and YL. Experimental design and execution, YL and HQ. Image collection, reading, and interpretation, HQ and CW. Data collection and analysis, HQ, CW, YT, JD, LL, and ZC. Manuscript editing, all authors. All authors contributed to the article and approved the submitted version.

## Funding

This work was supported by the National Natural Science Foundation of China (no. 81872396 and 61773134) and the National Undergraduate Innovation and Entrepreneurship Training Program (no. 202110226018).

## Conflict of Interest

The authors declare that the research was conducted in the absence of any commercial or financial relationships that could be construed as a potential conflict of interest.

## Publisher’s Note

All claims expressed in this article are solely those of the authors and do not necessarily represent those of their affiliated organizations, or those of the publisher, the editors and the reviewers. Any product that may be evaluated in this article, or claim that may be made by its manufacturer, is not guaranteed or endorsed by the publisher.
