# Supplementary material for: Predicting Chemo-Radiotherapy Sensitivity With Concordant Survival Benefit in Non-Small Cell Lung Cancer via Computed Tomography Derived Radiomic Features
Source: Front Oncol. 2022 Jun 22;12:832343. doi: 10.3389/fonc.2022.832343 (PMC9256940; doi:10.3389/fonc.2022.832343)
Supplement: Supplementary file 1 [file DataSheet_1.docx]

Supplementary Material

# Supplementary Figures and Tables

## Supplementary Figures


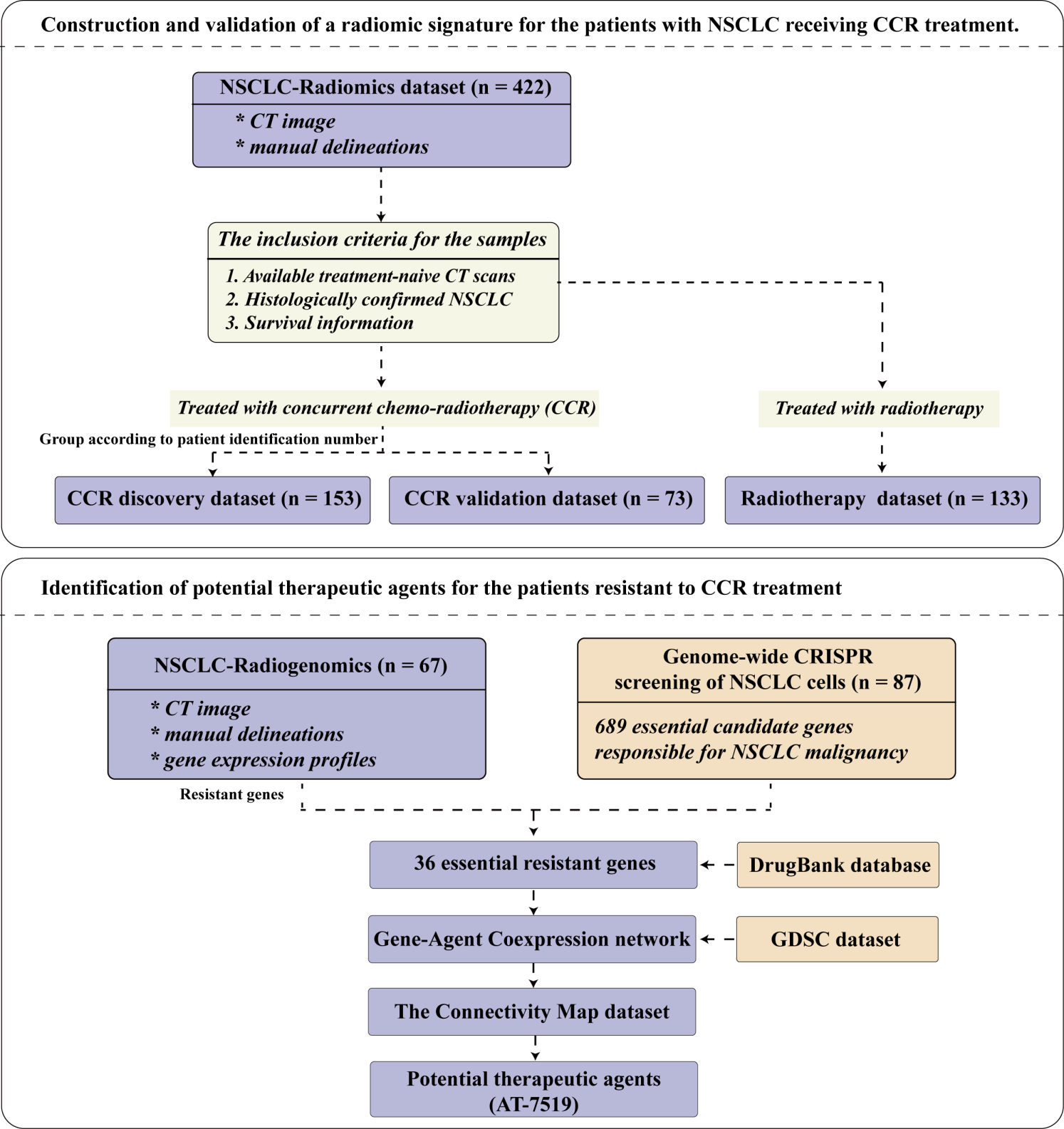


## Supplementary Figure S1. The details and applications of the analyzed datasets in this study.

##
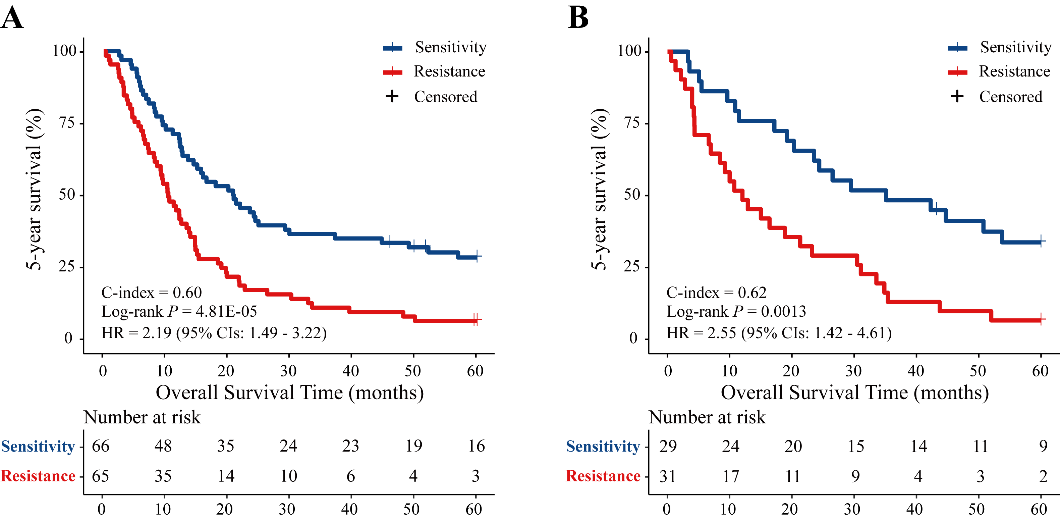


## Supplementary Figure S2. Kaplan–Meier curves of 5-year survival rate for patients with clarifying histologic subtypes. (A) Kaplan–Meier curves of 5-year survival rate for patients in the discovery dataset (*n*=131). (B) Kaplan–Meier curves of 5-year survival rate for patients in the validation dataset (*n*=60).

##
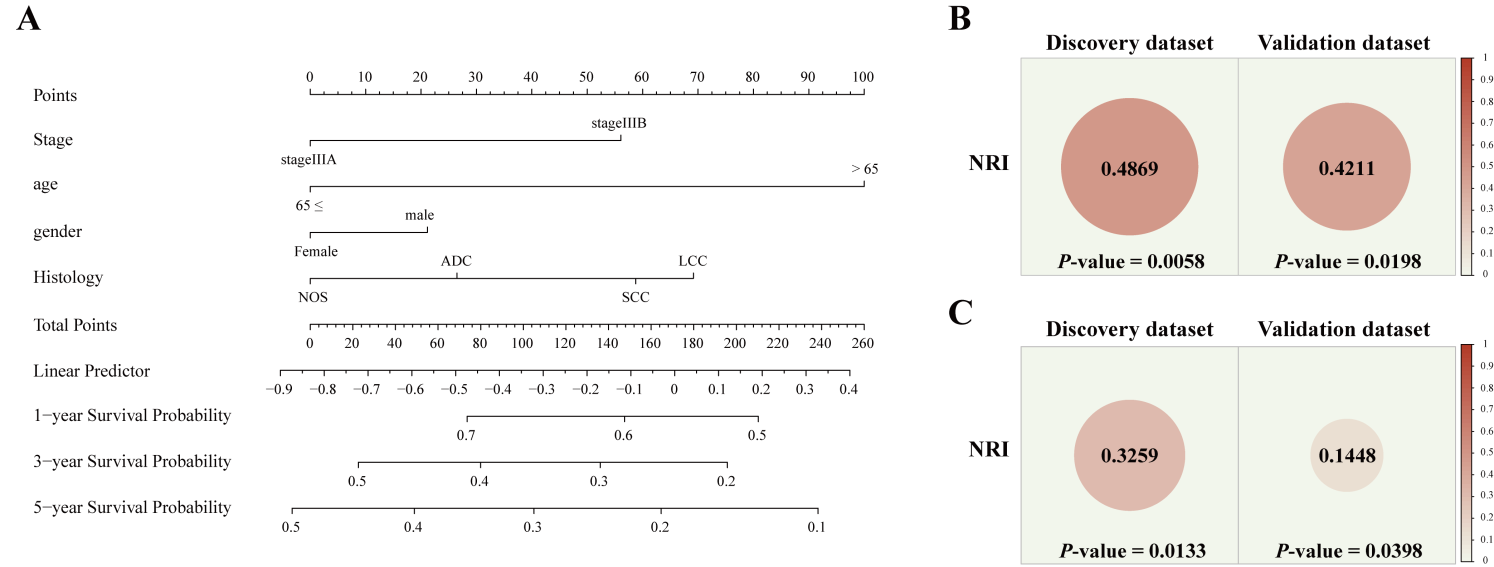


## Supplementary Figure S3. The clinical nomogram and performance improvement evaluation of the radiomic nomogram. (A) The clinical nomogram in the discovery cohort. (B) Performance improvement of the radiomic nomogram compared with clinical nomogram estimated by net reclassification improvement (NRI) method. (C) Performance improvement of the radiomic nomogram compared with CCR-9RS estimated by NRI method.

**
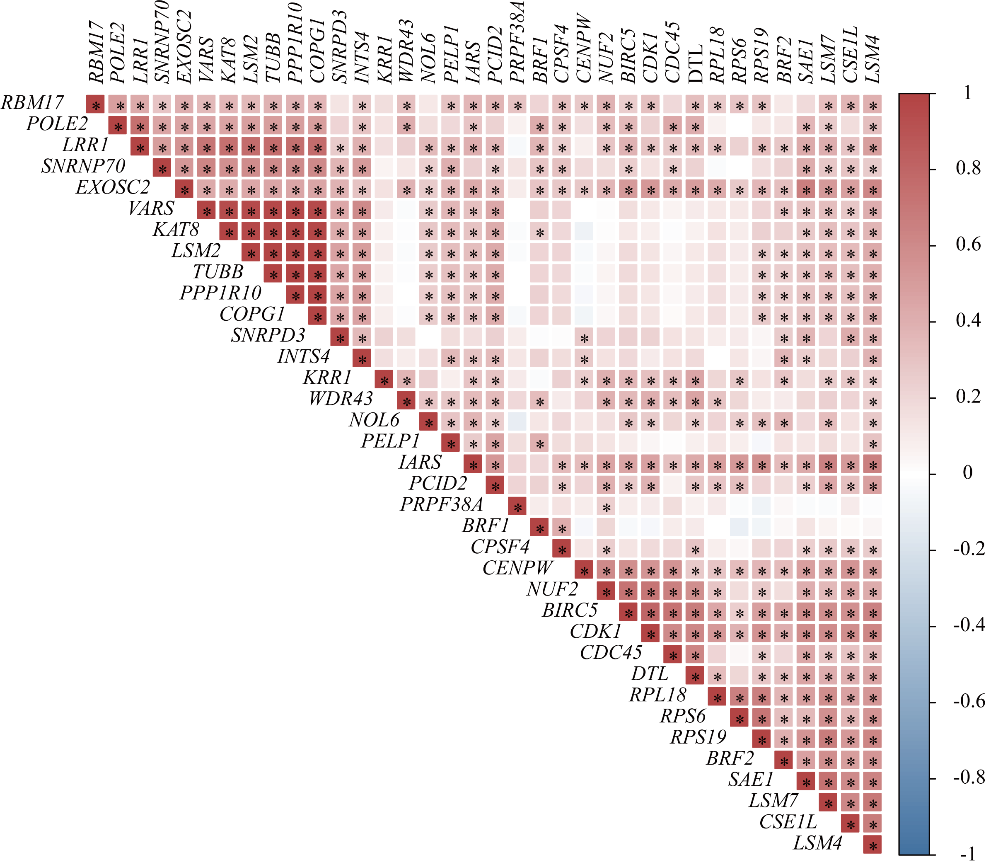
**

**Supplementary Figure S4.** **The correlations among the 36 essential** **resistant genes**. The responding significance is shown by asterisks in the squares (*for Pearson correlation, FDR < 0.05).

**
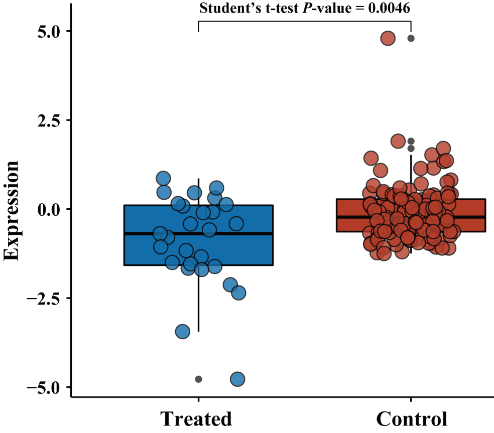
**

**Supplementary Figure S5.** **The boxplot of the *CDK1* gene which was significantly different between the AT-7519-treated and control groups in the CMap dataset.**

**
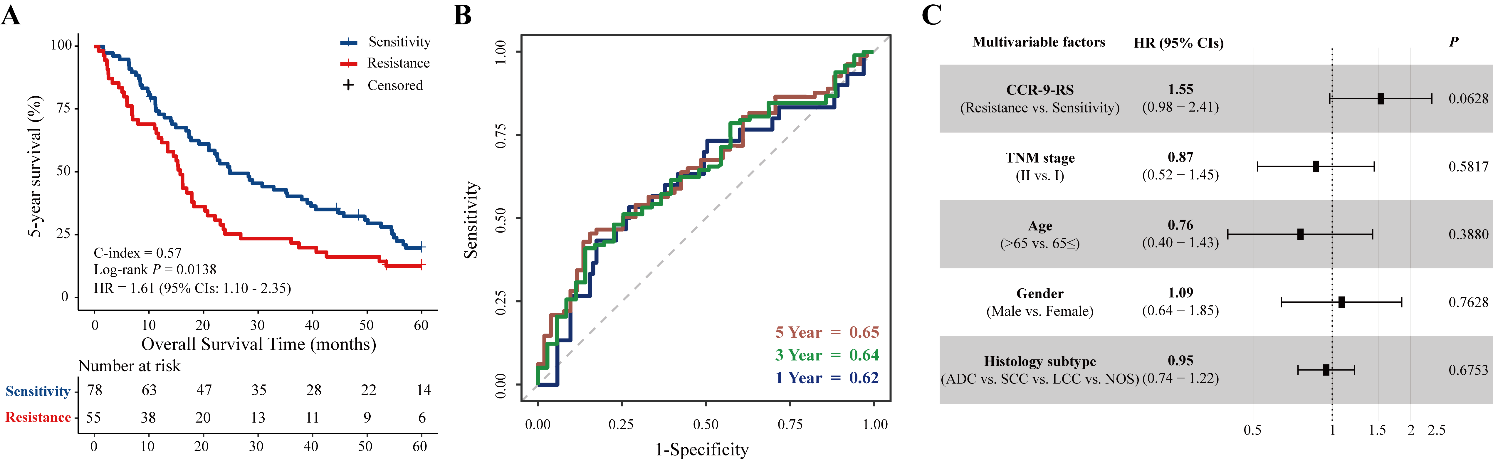
**

**Supplementary Figure S6. The survival analyzes** **for patients with NSCLC receiving radiotherapy.** (A) Kaplan–Meier curves of 5-year survival rate for patients in the radiotherapy dataset (*n* = 133). (B) Time-dependent receiver operating characteristic curve (ROC) of CCR-9RS in predicting 1-, 3- and 5-year survival rates in the radiotherapy dataset (C) Multivariate Cox analyzes of CCR-9RS after adjusting for clinical factors in the radiotherapy dataset.

**
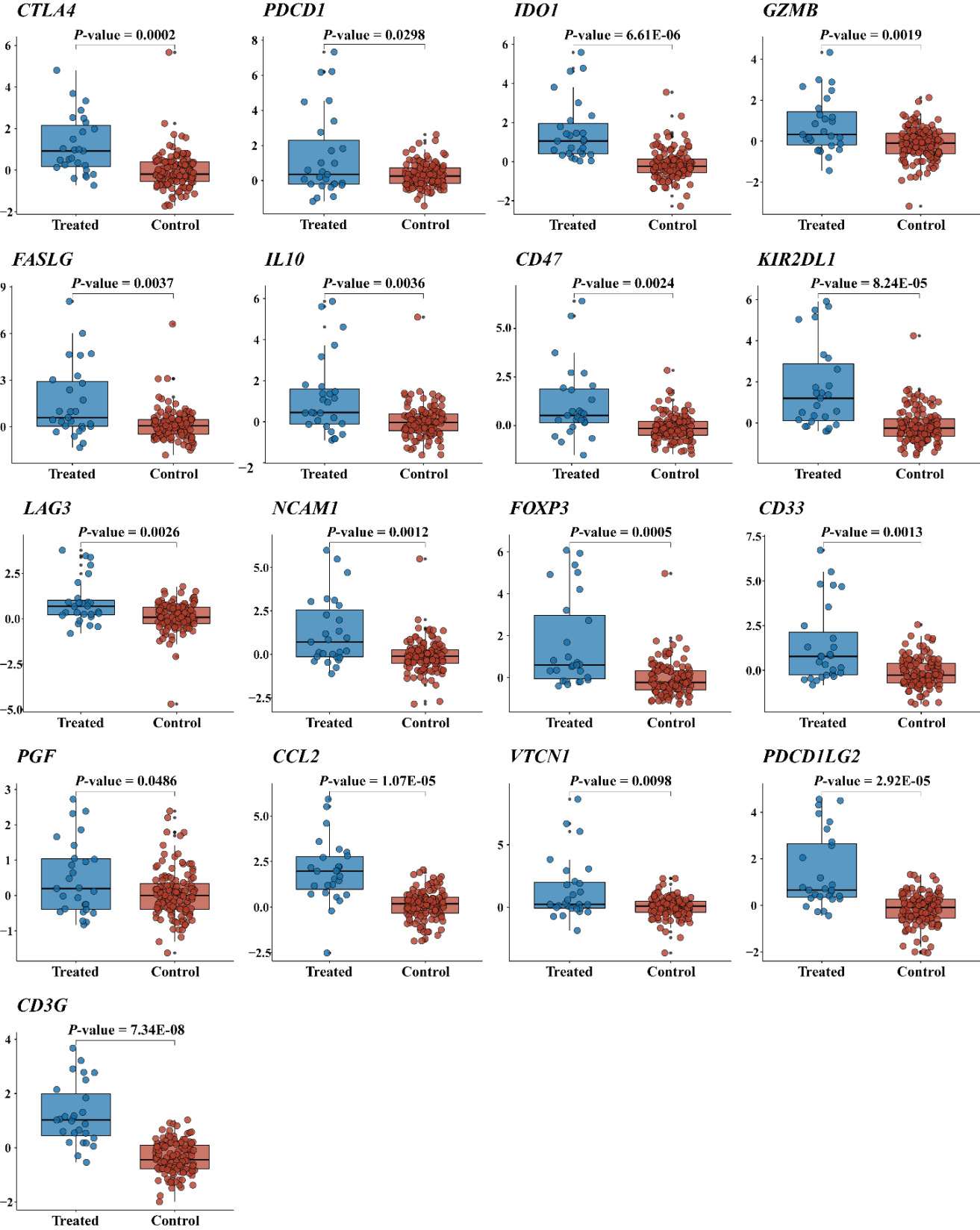
**

**Supplementary Figure S7. The boxplots of the expression levels of 17 immune inhibitor/checkpoint genes were significantly up-regulated in the AT-7519 treated samples compared with the untreated control samples in CMap dataset (Student’s *t* test, *P* < 0.05).**

## Supplementary Tables

**Supplementary Table S1. CT derived radiomic features for analysis in this study**

| **Type** | **Details** | **Number** |
| --- | --- | --- |
| Tumor intensity | InterquartileRange, Skewness, Uniformity, Median, Energy, RobustMeanAbsoluteDeviation, MeanAbsoluteDeviation, TotalEnergy, Maximum, RootMeanSquared, 90Percentile, Minimum, Entropy, Range, Variance, 10Percentile, Kurtosis, Mean | 18 |
| Shape | VoxelVolume, Maximum3DDiameter, MeshVolume, Elongation, MajorAxisLength, Sphericity, LeastAxisLength, SurfaceVolumeRatio, Maximum2DDiameterSlice, Flatness, SurfaceArea, MinorAxisLength, Maximum2DDiameterColumn, Maximum2DDiameterRow | 14 |
| Texture | Gray Level Co-occurrence Matrix (GLCM) | 24 |
|  | Gray Level Dependence Matrix (GLDM) | 14 |
|  | Gray Level Run Length Matrix (GLRLM) | 16 |
|  | Gray Level Size Zone Matrix (GLSZM) | 16 |
|  | Neighbouring Gray Tone Difference Matrix (NGTDM) | 5 |
| Wavelet filters | Features were calculated using wavelet filters. The value in brackets indicates the filters (H: High pass filter, L: Low pass filter) applied in the x, y and z directions, respectively. | 744 |
| Laplacian of Gaussian filters | Features were calculated using Laplacian of Gaussian filters. The value in brackets indicates the filter width used for the Gaussian kernel (1mm, 2mm, 3mm, 4mm, 5mm) | 465 |
| Logarithm filters | Features were calculated using Logarithm filters | 93 |
| Square filters | Features were calculated using Square filters | 93 |
| Exponential filters | Features were calculated using Exponential filters | 93 |
| Gradient filters | Features were calculated using Gradient filters | 93 |
| Squareroot filters | Features were calculated using Squareroot filters | 93 |

**Supplementary Table S2. Radiomic features and their weightings contributing to CCR-9RS**

| **Radiomic feature names** | **Coefficients** |
| --- | --- |
| squareroot_gldm_DependenceVariance | 0.01671323 |
| wavelet_LHH_glcm_JointAverage | 0.00551643 |
| wavelet_LHH_glcm_SumAverage | 0.00000013 |
| wavelet_LHH_firstorder_Range | 0.00002591 |
| wavelet_LHH_glszm_ZoneEntropy | 0.02806518 |
| wavelet_LLH_glrlm_LongRunHighGrayLevelEmphasis | 0.00000057 |
| wavelet_LLH_glszm_SizeZoneNonUniformity | 0.00034495 |
| wavelet_HHH_glszm_SizeZoneNonUniformity | 0.00134988 |
| wavelet_HHL_glszm_SizeZoneNonUniformityNormalized | 1.18176800 |

**Supplementary Table S3. Gene enrichment analysis of significantly correlated genes with radiomic features in CCR-9RS based on KEGG database**

| **Radiomic feature in CCR-9RS** | **KEGG pathway** | **FDR** | **Gene number (N vs. P)** |
| --- | --- | --- | --- |
| squareroot_gldm_DependenceVariance | Vascular smooth muscle contraction | 0.0005 | 25 (19 : 6) |
| squareroot_gldm_DependenceVariance | Oxytocin signaling pathway | 0.0077 | 26 (17 : 9) |
| squareroot_gldm_DependenceVariance | Circadian entrainment | 0.0077 | 17 (15 : 2) |
| squareroot_gldm_DependenceVariance | cGMP-PKG signaling pathway | 0.0092 | 25 (17 : 8) |
| squareroot_gldm_DependenceVariance | Dilated cardiomyopathy | 0.0163 | 16 (15 : 1) |
| wavelet_LHH_glcm_JointAverage | Ribosome | 1.83E-22 | 52 (0 : 52) |
| wavelet_LHH_glcm_JointAverage | Coronavirus disease - COVID-19 | 2.28E-12 | 49 (2 : 47) |
| wavelet_LHH_glcm_JointAverage | RNA transport | 0.0012 | 29 (4 : 25) |
| wavelet_LHH_glcm_JointAverage | Oxidative phosphorylation | 0.0251 | 20 (0 : 20) |
| wavelet_LHH_glcm_JointAverage | Huntington disease | 0.0454 | 36 (4 : 32) |
| wavelet_LHH_glcm_SumAverage | Ribosome | 1.83E-22 | 52 (0 : 52) |
| wavelet_LHH_glcm_SumAverage | Coronavirus disease - COVID-19 | 2.28E-12 | 49 (2 : 47) |
| wavelet_LHH_glcm_SumAverage | RNA transport | 0.0012 | 29 (4 : 25) |
| wavelet_LHH_glcm_SumAverage | Oxidative phosphorylation | 0.0251 | 20 (0 : 20) |
| wavelet_LHH_glcm_SumAverage | Huntington disease | 0.0454 | 36 (4 : 32) |
| wavelet_LHH_firstorder_Range | Ribosome | 3.57E-18 | 45 (0 : 45) |
| wavelet_LHH_firstorder_Range | Coronavirus disease - COVID-19 | 9.98E-07 | 37 (0 : 37) |
| wavelet_LHH_firstorder_Range | Oxidative phosphorylation | 0.0022 | 21 (0 : 21) |
| wavelet_LHH_firstorder_Range | Huntington disease | 0.0138 | 35 (1 : 34) |
| wavelet_LHH_firstorder_Range | Amyotrophic lateral sclerosis | 0.0244 | 38 (2 : 36) |
| wavelet_LHH_firstorder_Range | Protein processing in endoplasmic reticulum | 0.0253 | 24 (0 : 24) |
| wavelet_LHH_firstorder_Range | Phagosome | 0.0306 | 18 (0 : 18) |
| wavelet_LHH_glszm_ZoneEntropy | Ribosome | 3.24E-20 | 62 (0 : 62) |
| wavelet_LHH_glszm_ZoneEntropy | Coronavirus disease - COVID-19 | 2.55E-09 | 59 (0 : 59) |
| wavelet_LHH_glszm_ZoneEntropy | Oxidative phosphorylation | 5.76E-05 | 34 (0 : 34) |
| wavelet_LHH_glszm_ZoneEntropy | Amyotrophic lateral sclerosis | 0.0239 | 61 (0 : 61) |
| wavelet_LHH_glszm_ZoneEntropy | Thermogenesis | 0.0262 | 43 (0 : 43) |
| wavelet_LHH_glszm_ZoneEntropy | Protein export | 0.0262 | 10 (0 : 10) |
| wavelet_LHH_glszm_ZoneEntropy | Huntington disease | 0.0262 | 53 (0 : 53) |
| wavelet_LHH_glszm_ZoneEntropy | Protein processing in endoplasmic reticulum | 0.0363 | 36 (0 : 36) |
| wavelet_LHH_glszm_ZoneEntropy | Parkinson disease | 0.0363 | 43 (0 : 43) |
| wavelet_LHH_glszm_ZoneEntropy | Ubiquitin mediated proteolysis | 0.0417 | 32 (0 : 32) |
| wavelet_LHH_glszm_ZoneEntropy | Pathways of neurodegeneration - multiple diseases | 0.0417 | 70 (0 : 70) |
| wavelet_LHH_glszm_ZoneEntropy | RNA transport | 0.0417 | 34 (0 : 34) |
| wavelet_HHL_glszm_SizeZoneNonUniformityNormalized | Ribosome | 0.0009 | 32 (1 : 31) |
| wavelet_HHL_glszm_SizeZoneNonUniformityNormalized | Oxidative phosphorylation | 0.0009 | 28 (1 : 27) |
| wavelet_HHL_glszm_SizeZoneNonUniformityNormalized | Glycolysis / Gluconeogenesis | 0.0101 | 15 (2 : 13) |
| wavelet_HHL_glszm_SizeZoneNonUniformityNormalized | Phagosome | 0.0136 | 25 (0 : 25) |
| wavelet_HHL_glszm_SizeZoneNonUniformityNormalized | Vibrio cholerae infection | 0.0139 | 14 (1 : 13) |
| wavelet_HHL_glszm_SizeZoneNonUniformityNormalized | Protein processing in endoplasmic reticulum | 0.0219 | 32 (1 : 31) |
| wavelet_HHL_glszm_SizeZoneNonUniformityNormalized | Protein export | 0.0242 | 9 (0 : 9) |
| wavelet_HHL_glszm_SizeZoneNonUniformityNormalized | Huntington disease | 0.0256 | 45 (7 : 38) |

Note: N represents the number of genes in the pathway negatively correlated with a radiomic feature; *P* represents the number of genes in the pathway positively correlated with a radiomic feature. KEGG represents Kyoto Encyclopedia of Genes and Genomes.

**Supplementary Table S4. The detailed information of cell line samples treated with AT-7519**

| **CMap ID** | **Drug** | **Induced** **dose (**µ**M)** | **Induced** **time (hour)** | **Cell line** | **Dose group** |
| --- | --- | --- | --- | --- | --- |
| 685466 | AT-7519 | 0.01 | 24 h | A549 | Low |
| 857476 | AT-7519 | 0.03 | 24 h | A549 | Low |
| 579137 | AT-7519 | 0.04 | 24 h | HCC515 | Low |
| 547651 | AT-7519 | 0.04 | 24 h | A549 | Low |
| 743833 | AT-7519 | 0.04 | 24 h | A549 | Low |
| 802349 | AT-7519 | 0.08 | 24 h | A549 | Low |
| 550941 | AT-7519 | 0.12 | 24 h | HCC515 | Low |
| 587289 | AT-7519 | 0.12 | 24 h | A549 | Low |
| 1061540 | AT-7519 | 0.12 | 24 h | A549 | Low |
| 858490 | AT-7519 | 0.125 | 24 h | A549 | Low |
| 797323 | AT-7519 | 0.25 | 24 h | A549 | Low |
| 552685 | AT-7519 | 0.37 | 24 h | HCC515 | Low |
| 540037 | AT-7519 | 0.37 | 24 h | A549 | Low |
| 698467 | AT-7519 | 0.37 | 24 h | A549 | Low |
| 749870 | AT-7519 | 0.74 | 24 h | A549 | Low |
| 533559 | AT-7519 | 1.11 | 24 h | HCC515 | Middle |
| 564922 | AT-7519 | 1.11 | 24 h | A549 | Middle |
| 1065215 | AT-7519 | 1.11 | 24 h | A549 | Middle |
| 640998 | AT-7519 | 1.11 | 24 h | A549 | Middle |
| 687501 | AT-7519 | 2.22 | 24 h | A549 | Middle |
| 573327 | AT-7519 | 3.33 | 24 h | HCC515 | Middle |
| 578528 | AT-7519 | 3.33 | 24 h | A549 | Middle |
| 691523 | AT-7519 | 3.33 | 24 h | A549 | Middle |
| 587869 | AT-7519 | 10 | 24 h | HCC515 | High |
| 537044 | AT-7519 | 10 | 24 h | A549 | High |
| 1060886 | AT-7519 | 10 | 24 h | A549 | High |
| 872867 | AT-7519 | 10 | 24 h | A549 | High |
